# Effect of alkalized urine on renal calculi in patients with gout: a protocol for a placebo-controlled, double-blinded randomized controlled trial

**DOI:** 10.1186/s13063-021-05721-8

**Published:** 2021-10-26

**Authors:** Ertao Jia, Haiqiong Zhu, Hongling Geng, Yadong Wang, Li Zhong, Shangwen Liu, Feng Lin, Jianyong Zhang

**Affiliations:** 1 The Department of Rheumatology, Shenzhen Traditional Chinese Medicine Hospital, No. 1, Fuhua Road, Futian District, Shenzhen, 518033 Guangdong China; 2 The Department of Rheumatology, The fourth Clinical Medical College of Guangzhou University of Chinese Medicine, No.1, Fuhua Road, Futian District, Shenzhen, 518033 Guangdong China; 3 Shenzhen Affiliated Hospital of Nanjing University of Chinese Medicine, Nanjing, China; 4 The Department of Gynecology, Guangdong Provincial Hospital of Chinese Medicine, The Second Affiliated Hospital of Guangzhou University of Chinese Medicine, Guangzhou, China; 5 The Department of Urology, Shenzhen Traditional Chinese Medicine Hospital, Shenzhen, China

**Keywords:** Gout, Renal calculus, Urine alkalization, Sodium bicarbonate

## Abstract

**Background:**

The prevalence of renal calculi in patients with gout is high. Alkalized urine has been recommended by the 2020 European Association of Urology (EAU) guidelines to promote calculus dissolution. However, randomized controlled trials are lacking.

**Methods:**

In the protocol of this randomized, placebo-controlled, double-blinded trial, patients with gout combined with renal calculi are randomized (1:1) to the placebo and sodium bicarbonate groups. The intervention would be performed for 24 weeks, the 1–12 weeks are double-blinded, and the 13–24 weeks are open-labeled. Sodium bicarbonate (1 g tid) will be performed for 24 weeks in the sodium bicarbonate group. The placebo will be performed for 12 weeks and not be performed from 13 weeks to 24 weeks in the placebo group. All subjects will be administered febuxostat (40 mg/day) for 24 weeks and receive concomitant anti-inflammatory prophylaxis therapy for 12 weeks. The primary outcome is the proportion of patients whose renal calculus volume will be reduced after 12 weeks of treatment. The secondary outcomes include the volume changes of renal calculi, uric acid changes, the proportion of patients with serum uric acid (sUA) levels < 360 μmol/L, the changes in estimated glomerular filtration rate (eGFR), the pH value of urine, and the incidence of adverse events after treatment for 12 and 24 weeks.

**Discussion:**

This study will evaluate the efficacy and safety of sodium bicarbonate-alkalized urine on renal calculi in patients with gout.

**Trial registration:**

ClinicalTrials.gov ChiCTR2100045183. Registered on April 7, 2021, with ChiCTR.

## Administrative information

**Table:** 

Title {1}	Effect of alkalized urine on renal calculi in patients with gout: a protocol for a placebo-controlled, double-blinded randomized controlled trial
Trial registration {2a and 2b}.	ClinicalTrials.gov ChiCTR2100045183. Registered on April 7, 2021, with ChiCTR http://www.chictr.org.cn/showproj.aspx?proj=124742
Protocol version {3}	April 23, 2021, V.20210423
Funding {4}	This study has been funded by the Shenzhen Science and Technology Plan Project (JCYJ20170817094922513), Sanming Project of Medicine in Shenzhen (SZSM201612080), and Shenzhen Science and Technology Plan Project (JCYJ20180302173532311).
Author details {5a}	EJ, JZ, and FL designed the randomized placebo-controlled trial. EJ, HG, and HZ drafted the manuscript. YW, LZ, SL, and JZ conducted the research. EJ was responsible for the statistical analyses. All authors participated in the manuscript revision.
Name and contact information for the trial sponsor {5b}	NA
Role of sponsor {5c}	NA

## Introduction

### Background and rationale {6a}

Gout is caused by disorders of purine metabolism, increased uric acid production, and/or decreased uric acid excretion, leading to elevated sUA levels, forming monosodium urate (MSU) crystals that are deposited in the joints and kidney and other tissues. A meta-analysis reported that the pooled prevalence of gout was 1.1% in China [1].

Nephrolithiasis is common in patients with gout. It has been showed that 24% of patients with gout are suffering from nephrolithiasis. The odds ratio (OR) of gout as a risk factor for nephrolithiasis was 2.4 1 [2], and the hazard ratio (HR) of renal calculus as a risk factor for the development of end-stage renal disease (ESRD) was 2.34–6.18 [3]. More than half of the patients with nephrolithiasis carried bilateral and multiple calculi [4]. The formation of renal calculi in patients with gout is mainly related to high level of uric acid. A previous study analyzed uric acid composition in gout patients by DECT and found that the proportion of pure and mixed uric acid in renal calculus was 77.8% [5]. The ionized forms of uric acid readily form salts, namely monosodium urate, disodium urate, or potassium urate. Sodium is the dominant cation in the extracellular fluid. It is estimated that 98% of uric acid forms monosodium urate at pH=7.4, and the saturation of monosodium urate in the human plasma is about 7 mg/dL. As urine is acidified along the renal tubules, a portion of urate is converted to uric acid. The solubility of uric acid in aqueous solution is less than that of urate, but the saturation increases significantly with the increase in the pH value of urine. At pH 5, the urinary saturation of uric acid is 15 mg/dL, while the value reaches 158–200 mg/dL at pH7, with an increase of uric acid solubility by 20-fold [5]. Gout patients with long-term high uric acid levels also have elevated urinary uric acid concentration and form crystals after exceeding the solubility, gradually enlarged to shape calculus [6]. Previous studies have shown that the course of gout and low pH value of urine [5] are the primary hazard factors of renal calculus.

Another study suggested that the pH value of urine increased from 5.5 to 6.0 by administering sodium bicarbonate (1 g tid) for 3 months in gout patients [7]. Although several urological associations recommended urine alkalization to promote calculi dissolution in nephrolithiasis [8–11], advanced evidence is lacking. Since alkaline urine may increase the risk of hypertension, water-sodium retention, and heart failure, according to the 2020 American College of Rheumatology (ACR) guideline, starting alkalizing urine is not recommended even in cases of stimulated uric acid excretion. Based on the composition, renal calculus is mainly divided into calcified calculi, urate calculi, cystine calculi, and combinations. Typically, nephrolithiasis is assessed by ultrasonography, plain film radiography, tomography, or excretory urography with an intravenous contrast agent [12]. However, none of these methods could identify calculi before treatment. Dual-energy computed tomography (DECT) is a novel non-invasive technique that fundamentally distinguishes the composition of urate calculi. This method has been previously validated in both in vitro and in vivo studies [12–15]. Moreover, it can also be used to calculate the volume of renal calculi. The present study aimed to determine the effect of sodium bicarbonate-alkalized urine on renal calculus in patients with gout.

### Objectives {7}

The present study aims to determine the effect of sodium bicarbonate-alkalized urine on renal calculus in patients with gout.

### Trial design {8}

**Fig. 1:**
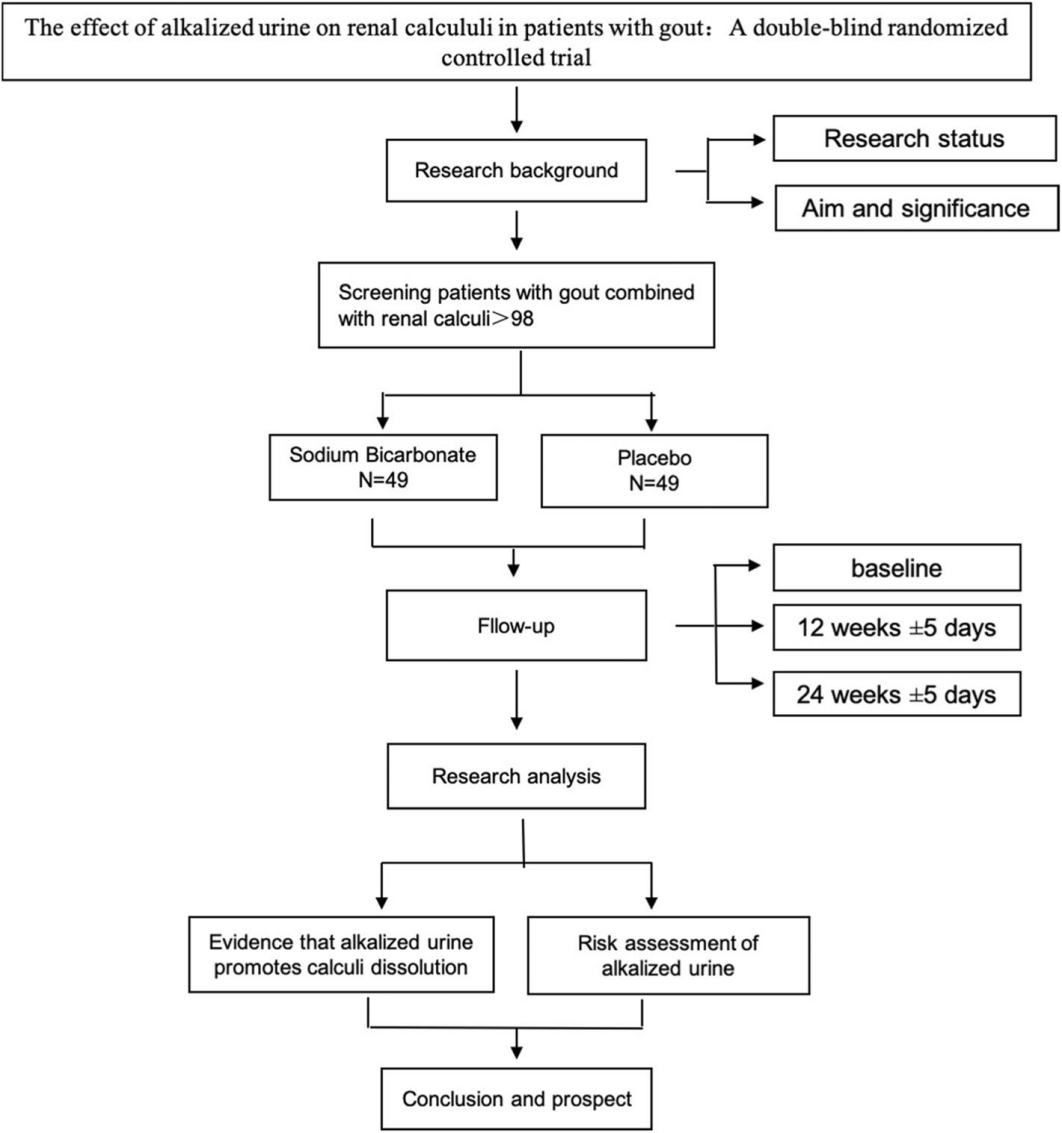
Trial flow and study design

This 12-week dual-center, double-blinded, placebo-controlled and two-arm randomized trial (RCT) will be completed at the Shenzhen Traditional Chinese Medicine Hospital and People’s Hospital of Longhua District Shenzhen. A total of 98 patients with gout combined with renal calculi who meet the study criteria will be randomized (1:1) to the placebo and sodium bicarbonate group. The intervention would be performed for 12 weeks with a follow-up of 24 weeks. The 1–12-week group is double-blinded, and the 13–24-week group is open-labeled (Fig. 1).

## Methods: participants, interventions, and outcomes

### Study setting {9}

This study will be completed at the Shenzhen Traditional Chinese Medicine Hospital and People’s Hospital of Longhua District Shenzhen from April 30, 2021, to April 30, 2024.

### Eligibility criteria {10}

#### Inclusion criteria

Participants who meet the following criteria will be included:

(1) Male and female patients aged 18–80 years old.

(2) The diagnosis meets the 2015 European League Against Rheumatism (ELAR)/ACR criteria for acute arthritis of gout.

(3) B-ultrasound suggesting renal calculi.

(4) Male with no fertility requirement in recent 6 months.

(5) Female who have undergone sterilization operation or have been menopausal for 2 years.

#### Exclusion criteria

Potential subjects will be excluded if they meet any of the following:

(1) Allopurinol, probenecid, benzbromarone, and febuxostat tablets have been used for urate-lowering treatment (ULT) in the first 4 weeks of enrolment.

(2) Renal calculus >15 mm, obstruction (hydronephrosis), pain, and urinary tract infections requiring lithotripsy [10].

(3) Patients with secondary hyperuricemia caused by kidney disease, hematopathy, some medications, tumor chemoradiotherapy, and organ transplantation.

(4) Patients with a history of cardiocerebrovascular diseases, such as stroke, TIA, MI, and HF (NYHA II-IV), coronary artery surgery (for example, angioplasty, stent implantation, and bypass grafting).

(5) Patients with histories of peptic ulcer and gastrointestinal bleeding.

(6) Patients with active stage of liver disease and abnormal liver function or with a transaminase level of 1.2-fold higher than the upper limit of normal value.

(7) Patients with abnormal renal function and eGFR <30 mL/min.1.73 m^2^.

(8) Patients with malignant tumor or psychosis.

(9) Patients being allergic or intolerant to febuxostat.

(10) Patients with other (non-gout) chronic arthritis, acute inflammatory arthritis, and autoimmune diseases associated with arthritis.

(11) Patients who take or need azathioprine, mercaptopurine, theophylline, and cytotoxic chemotherapeutic drugs.

(12) Patients with a history of alcohol or drug dependence or need long-term daily painkillers for any reason.

(13) Patients who have been involved in other clinical investigations within the first 3 months of enrolment.

#### Who will take informed consent? {26a}

Each subject will obtain informed consent or assent from potential trial participants or authorized surrogates.

#### Additional consent provisions for the collection and use of participant data and biological specimens {26b}

Not applicable.

### Interventions

#### Explanation for the choice of comparators {6b}

A total of 98 patients with gout combined with renal calculi who meet the study criteria will be randomized (1:1) to the placebo and sodium bicarbonate group.

#### Intervention description {11a}

The intervention would be performed for 24 weeks, the 1–12 weeks are double-blinded, and the 13–24 weeks are open-labeled. The volume of renal calculi is slowly decreases and 24 weeks may be needed, while this will lead to the increasing rate of shedding, so the 13–24 weeks are open-labeled.

(1) Sodium bicarbonate group: sodium bicarbonate (1 g tid) will be performed for 24 weeks.

(2) Placebo group: the placebo will be performed for 12 weeks and not be performed from 13 weeks to 24 weeks.

(3) Both groups are administered febuxostat (40 mg/day) for 24 weeks.

(4) Drugs to prevent gout flares: prednisone acetate (5 mg Qd), celecoxib, or colchicine (12 weeks).

(5) During this trial, all subjects will receive general treatment, including ① strict diet: intake of low-calorie diet to maintain ideal body weight. Avoid food with high purine content, such as animal offal, thick gravy soup, sardines, and clams, oysters. The moderate consumption of fish, shrimp, meat, and pea with a moderate amount of purine. The daily diet consists of low purine content of cereal products, fruits, vegetables, milk, dairy products, and eggs. Also, drinking >2000 mL of water every day and give up all kinds of alcohol strictly. ② Avoid inducements including smoking, overeating, cold, dampness, overfatigue, and stress. In addition, it is necessary to slip into comfortable shoes, prevent damage of joint, and only sparingly use drugs that seriously affect uric acid excretion (such as some diuretics). ③ Concomitant disease control: concurrent treatment of concomitant hyperlipidemia, diabetes, hypertension, coronary heart disease, and cerebrovascular disease.

#### Research procedures

(1) Screening: In line with 2015 ACR and EULAR criteria and the score system for acute arthritis of gout, B ultrasonography suggests renal calculi. The patients are 18–80 years old.

(2) Enrolment: Each subject will be interviewed by investigators prior to the start of the treatment, and the data would be input into the database along with baseline data. The subjects will be divided into test and control groups according to the treatment, and both groups will be treated for 6 months. Renal calculus volume changes before and after treatment will be observed and recorded, respectively.

(3) Treatment scheme: Test group: sodium bicarbonate 1 g tid combined with febuxostat 40 mg/day and prednisone acetate 5 mg Qd for 12 weeks. Control group: placebo 1 g tid combined with febuxostat 40 mg/day and prednisone acetate 5 mg Qd for 12 weeks.

(4) Laboratory indicators: Vital signs (pulse, blood pressure, temperature, respiration, and gout flares), four physiological indicators of blood lipids (TC, TG, HDL-C, LDL-C), fasting blood glucose, blood routine (RBC, WBC, NEUT, PLT, Hb), urine routine (pH value of urine, PRO, BLD), renal function (BUN, CREA, sUA, EGFR), liver function (ALT, AST, GGT, ALP), erythrocyte sedimentation rate (ESR), C-reactive protein (CRP), and renal DECT (location, size, quantity component of renal calculi).

#### Criteria for discontinuing or modifying allocated interventions {11b}

Rejection criteria

(1) The combination of drugs violates the protocol, or patients fail to use drugs in accordance with the provision, which in turn affects the outcomes.

(2) Incomplete data affecting the efficacy and the judgment of safety.

Shedding criteria

(1) Serious adverse reactions related to the study drug.

(2) In this study, major mistakes are detected in the clinical research protocol, which makes it difficult to evaluate the efficacy of the drug. Also, a significant deviation is observed in the implementation of a well-designed protocol.

Subject termination criteria

(1) Subject terminates spontaneously (for example, withdrawal of the informed consent).

(2) Subject is pregnant (must drop out of the study).

(3) Subject uses a combination of drugs within a non-prescribed range, which affects the judgment of efficacy and safety.

(4) Indicators of liver function: transaminase ≥3 times the upper limit of normality for more than 1 week.

(5) Adverse events that prompts the investigator to decide that the subject has to quit early.

(6) Compliance of the subject with the research protocol is poor, and the quantity of medication is not between 80% and 120%.

#### Strategies to improve adherence to interventions {11c}

We will call the subjects back to the hospital regularly for follow-up, laboratory tests were performed, and subjects were asked to return the drug tablet at each follow-up visit.

#### Relevant concomitant care permitted or prohibited during the trial {11d}

*This* trial will not impose *special requirements for* care and interventions.

#### Provisions for post-trial care {30}

When the trial complete, the subjects will receive standardized treatment based on the guideline.

#### Outcomes {12}

##### Primary outcomes

The proportion of patients whose renal calculus volume is reduced after 12 weeks of treatment.

##### Secondary outcomes

The volume changes of renal calculi, uric acid changes, and the proportion of patients whose sUA levels are <360 μmol/L, the changes in eGFR, the pH value of urine, and adverse effects after 12 and 24 weeks of treatment. Adverse events will be monitored during the 24 weeks of treatment, including the incidence of hypertension before and after treatment, the incidence of post-treatment edema, and the rate of acute gout attack within 12 weeks. Any adverse reactions that occur during the study will be recorded in the “Adverse Reaction Table,” and the patients will be followed up until symptoms disappear or indicators return to normal. In the event of serious adverse events, necessary measures will be taken immediately to ensure the safety of the subjects. In addition to evaluating the primary and secondary outcomes, safety assessment will be conducted at baseline and at 24 weeks after treatment with respect to blood pressure, respiration, heart rate, blood routine, urine routine, liver and renal function, ESR, CRP, and renal DECT.

#### Participant timeline {13}

See Fig. 2.

**Fig. 2:**
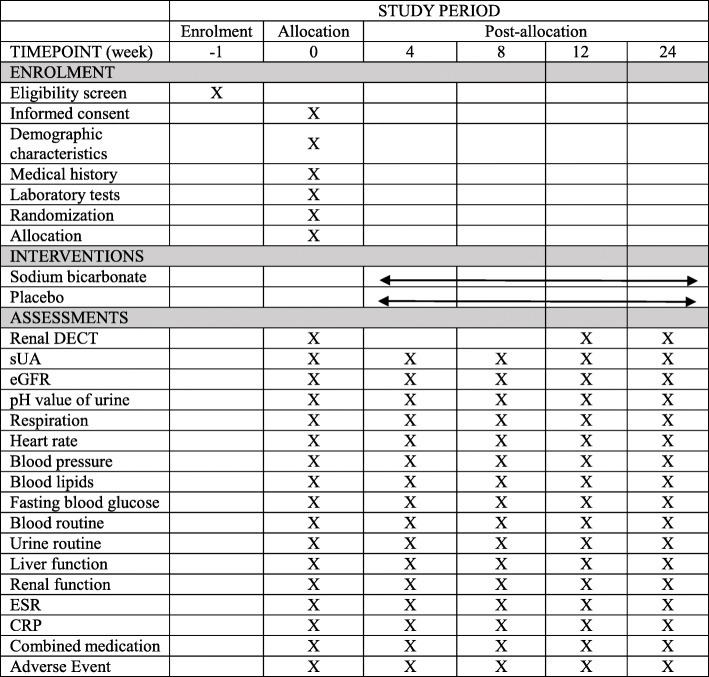
SPIRIT figure of enrolment, interventions, and assessments. DECT dual-energy-computed tomography, sUA serum uric acid, eGFR estimated glomerular filtration rate, ESR erythrocyte sedimentation rate, CRP C-reactive protein

#### Sample size {14}

This is a differential test, and the primary outcome is the proportion of patients whose renal calculus volume is reduced after 3 months of treatment. A test level with a two-sided *P* value of 0.05 and a power of 80% is assumed, and the ratio of patients’ number in the sodium bicarbonate to the placebo group was 1:1. The parameters of preliminary studies have demonstrated that the reduction rate of renal calculus volume in the test group is 89.74% in 3 months and 64.10% in the control group. The estimated sample size will be 42 in the trial group and 84 in the control group. In this study, the shedding rate does not exceed 15%, and the sample size for each group is determined to be 49.

#### Recruitment {15}

The randomized controlled trial that is blinded to assessors and patients will be conducted at the Shenzhen Traditional Chinese Medicine Hospital and People’s Hospital of Longhua District Shenzhen, wherein medical service would be provided for patients with gout combined with renal calculi. The subjects in this study will be recruited from outpatient and inpatient by placing advertisements on social media platforms of the hospital departments and distributing posters in public areas of the hospital with details of the study and contact information.

Subjects interested will be welcome to contact the investigators by phone or on-site for initial screening. Potential participants would be enrolled, and an appointment was made with a designated physician to examine the inclusion and exclusion criterion. Patients who meet the criteria will be invited to participate in the study, and details of the RCT protocol will be provided. All subjects will be required to sign an informed consent and provide complete information. The subjects will be randomized (1:1) to the placebo and sodium bicarbonate group, and none of the patients would know about their assigned group. Then, the baseline characteristics will be assessed and a database record is obtained by the same physician. All subjects will be treated for 24 weeks and followed up on 4, 12, and 24 weeks, and the data will be evaluated. In addition, all subjects will be given a schedule of intervention dates and follow-up appointments. These evaluations will be performed by an assistant who is involved neither in the randomization nor in the treatment.

#### Assignment of interventions: allocation

##### Sequence generation {16a}

The statistician used the PROC PLAN process of SAS 9.4 statistical software to generate random numbers with block randomization of variable block length.

##### Concealment mechanism {16b}

The mechanism of implementing the allocation sequence; Dr. Zhiying Zhan generates the allocation sequence, sequentially numbered, opaque, sealed envelopes, and can be reproduced when needed.

##### Implementation {16c}

Dr. Zhiying Zhan (Fujian Medical University School of Public Health) generates the allocation sequence, the doctors will enrol participants, and the nurse will assign participants to interventions.

#### Assignment of interventions: blinding

##### Who will be blinded {17a}

A total of 98 eligible subjects will be randomly assigned to two parallel groups. Statisticians adopt the PROC PLAN process of SAS 9.4 statistical software to generate a random list by randomization of variable block lengths. Then, the clinical trial manager divided them into two groups according to the random list. All people involved including the investigators are blinded to the assignment of the subjects. The placebo is similar to sodium bicarbonate in size, weight, shape, and color. Doctors, subjects, and data analysts are unclear about the type of drug. The randomized assignment sequence is placed in a sealed opaque envelope, and the blind codes are kept in the research management department and can be reproduced when needed.

##### Procedure for unblinding if needed {17b}

Unblinding meetings should be held for whatever reason firstly. Unblinding is realized by connecting offline password letters with e-mail. *Investigator*s will open offline letters to obtain the unblind password, log into the website of unblind system online in non-account login mode directly, and then fill in the relevant information (e.g., subject number, unblinded password) as prompted on the page. After input, the system can associate the electronic letter corresponding to the subject number with the unblinded password and open it to obtain the blind information. The treatment group that subject received will then be displayed on the web page. At the same time, the system will automatically record all the relevant information of the unblinding and inform relevant personnel by message. The code is invalid once used.

#### Data collection and management

##### Plans for assessment and collection of outcomes {18a}

The Case Report Form is used to assess, collect outcome, and baseline. In addition, the clinical trial database is constructed by a designated data manager who is responsible for the regular database management and maintenance. All data will be imported into the clinical trial database by two research assistants.

##### Plans to promote participant retention and complete follow-up {18b}

Follow-up will be conducted at baseline, 4 weeks ± 3 days, 8 weeks ± 3 days, 12 weeks ± 3 days, and 24 weeks ± 3 days. Any missing or incorrect data will be detected by software system. In such case, the original CRFS will be checked to correct or complete every piece of data.

##### Data management {19}

The clinical trial data are managed by Zhiying Zhan (Fujian Medical University School of Public Health) to ensure the authenticity, integrity, and privacy of the data during the research process. The clinical trial database is constructed by a designated data manager who is responsible for the regular database management and maintenance. All data will be imported into the clinical trial database by two research assistants.

##### Confidentiality {27}

The confidentiality measures are as follows. The results of this research project may be published in medical journals. The subject’s information is represented by a unique number, and the coded information is stored in the school of Public Health, Fujian Medical University. The information of subjects will be maintained confidential as required by law. However, records of subjects may be reviewed to ensure that the study complies with applicable laws and regulations.

##### Plans for collection, laboratory evaluation, and storage of biological specimens for genetic or molecular analysis in this trial/future use {33}

Not applicable.

### Statistical methods

#### Statistical methods for primary and secondary outcomes {20a}

The primary outcome will be the comparison between the groups using Pearson’s chi-square test. *t* test, corrected *t* test (equal variance not assumed), and analysis of variance (ANOVA) for repeated measurements will be used for data analysis, and the grade data will be assessed by Wilcoxon two-sample test.

#### Interim analyses {21b}

Doctors will have access to these interim results and make the final decision to terminate the trial.

#### Methods for additional analyses (e.g., subgroup analyses) {20b}

The hybrid control will use multivariate logistic regression, estimating the odds ratio (OR), and 95% confidence interval (CI). Clinically, significant variables in the univariate analysis will be included in the multivariate model. The goodness of fit will be evaluated by Hosmer–Lemeshow test. The statistical analysis was carried out using SPSS (version 26). A two-tailed significance level of 0.05 was used for all tests. *P*<0.05 indicated statistical significance.

#### Methods in the analysis to handle protocol non-adherence and any statistical methods to handle missing data {20c}

Study populations include the intent-to-treat (ITT) analysis set defined as all randomized patients, and the per-protocol (PP) analysis set defined as all patients in the ITT population without any major protocol deviations. Multiple imputation is used to manage missing values.

#### Plans to give access to the full protocol, participant level-data, and statistical code {31c}

Data are available upon reasonable request. For inquiries about data sharing, please send a request to sailing1980@126.com.

### Oversight and monitoring

#### Composition of the coordinating center and trial steering committee {5d}

The trial steering committee consists of three members, two senior rheumatologists, and a statistician, who will oversee the trial. The committee is independent of the research team, and there is no conflict of interest. Information on the composition, roles, and responsibilities of group providing day-to-day support to the trial is provided below. The trial was designed by a senior rheumatologist. A rheumatologist and two urologists collect clinical data.

#### Composition of the data monitoring committee, its role, and reporting structure {21a}

The data monitoring committee consists of three members, two senior rheumatologists, and a statistician, who will ensure the safety and quality of data. The committee is independent of the research team, and there is no conflict of interest. They will provide regular supervision, hold monthly meetings, and organize a field trip at least once to ensure the trial is carried out smoothly and ethically. Also, a supervisor will ensure the authenticity and integrity of the data. During the visit, they will interview the investigators, check the original research documents and subject registration, and confirm whether the clinical centers comply with the research protocol. Any non-compliance with the agreement will be fully recorded using a violation report form.

#### Adverse event reporting and harms {22}

The drugs used in the study were recommended in the guidelines for diagnosis and treatment of gout/hyperuricemia and its drug therapies will lead to hepatic impairment. Certain patients are administered sodium bicarbonate may be reactions to the gastrointestinal symptoms. Prednisone acetate induces osteoporosis and potentially increasing the risk of subsequent infections.

Symptomatic treatment would be initiated for you if there is an adverse event during the treatment. In case of hepatic impairment, hepatoprotective therapy was administered (e.g., compound glycyrrhizin for injection, silymarin). Certain patients are administered compound azintamide enteric-coated tablets to prevent gastrointestinal side effects caused by sodium bicarbonate. Prednisone acetate induces osteoporosis and potentially increasing the risk of subsequent infections. Once infected, it is necessary to immediately identify the nature of infection, select sensitive drugs in order to rapidly reach the steady range, and at the same time withdraw the dosage of glucocorticoid.

#### Frequency and plans for auditing trial conduct {23}

We will audit the trial conduct every 3 months.

##### Plans for communicating important protocol amendments to relevant parties (e.g., trial participants, ethical committees) {25}

Committees will identify the problems in the trial and put forward suggestions on the modification of the protocol. If any decision to amend the protocol has to be made, a written application needs to be submitted to the Institutional Medical Ethics Committee, and the investigator will be notified in writing after approval. The protocol will be updated immediately in the system.

##### Dissemination plans {31a}

All presentations should protect the integrity of the primary research objectives. Any data that compromises the blinding will not be released before the results are available. The steering committee will discuss the recommendations on the timing of these final data that may be presented at the meetings. The primary outcomes will be published in abstract books and articles.

## Discussion

In patients with gout, vascular injury caused by hyperuricemia and the deposition of urate crystals in the medulla is a major cause of substantial kidney damage, increasing the risk of chronic kidney disease and thus exerting a significant impact on the living quality of patients. Xanthine oxidase inhibitors alone are not as effective as alkalizing urine drugs in preventing renal calculi. Commonly used drugs include sodium bicarbonate and potassium sodium hydrogen citrate. However, both drugs give rise to adverse reactions, resulting in poor patient compliance [16]. Furthermore, recommendations by the 2020 ACR and EAU guidelines for the therapy of renal calculi in gout patients by alkalizing urine are controversial [10, 11]. Due to the lack of strong evidence, we have designed a RCT to analyze the effect of urine alkalization on renal calculi in gout patients by evaluating whether the renal calculus volume is reduced after 12 weeks of treatment. This study will provide guidance on the use of accurate medical methods such as urine alkalization for patients, thereby reducing the incidence of gout flares.

Nevertheless, the present study has some limitations. First, the universality of the sample population and the research centers involved in the trial is limited. In order to complete this clinical trial successfully, the selected participants should be local residents from Shenzhen, which will ensure their participation throughout the treatment. Patients with gout combined with renal calculi under investigation may not have urate calculi, as identified by DECT. In addition, the EAU guideline recommends administration of allopurinol during ULT, requiring the sUA level of each case meeting the standard (> 4.0 mmol/day and/or > 380 μmol/L) [11]. Previous studies have shown that gout patients have a high rate of sUA less than 360 μmol/L who have been treated with febuxostat (40 mg/d) in China [17]. Taken together, this clinical trial aims to explore if urine alkalization is suitable for patients with gout combined with renal calculi and whether the associated adverse events can be avoided. Finally, we hope that clinicians and gout patients can focus on the treatment of alkalized urine for renal calculi.

### Trial status

The revised version V20210423 of this protocol on April 23, 2021, has been approved by the Institutional Medical Ethics Committee of Shenzhen Traditional Chinese Medicine Hospital and People’s Hospital of Longhua District Shenzhen. The recruitment will begin on July 1, 2021, and this study will be completed on June 30, 2023.

## Abbreviations

RCT: Randomized controlled trial
ACR: American College of Rheumatology
EULAR: European League Against Rheumatism
MSU: Monosodium urate crystals
ESRD: End-stage renal disease
DECT: Dual-energy computer tomography
OR: Odds ratio
HR: Hazard ratio
CI: Confidence interval
ULT: Urate-lowering treatment
eGFR: Estimated glomerular filtration rate
sUA: Serum uric acid
ESR: Erythrocyte sedimentation rate
CRP: C-reactive protein
ITT: Intent-to-treat
PP: Per-protocol
ANOVA: Analysis of variance
